# AV timing in pacemaker patients with first-degree AV block: which is preferable, intrinsic AV conduction or pacing?

**DOI:** 10.1007/s00380-022-02037-8

**Published:** 2022-02-08

**Authors:** Yoshihiro Aizawa, Toshiko Nakai, Yukitoshi Ikeya, Rikitake Kogawa, Yuki Saito, Kazuto Toyama, Tetsuro Yumikura, Naoto Otsuka, Koichi Nagashima, Yasuo Okumura

**Affiliations:** Department of Medicine, Division of Cardiology, Nihon University School of Medicine, 30-1 Ohyaguchi-kamicho, Itabashi-ku, Tokyo, 173-8610 Japan

**Keywords:** Long atrioventricular conduction, Right ventricular pacing, Heart failure, Optimal atrioventricular delay, Diastolic function

## Abstract

Some patients with pacemakers present with first-degree atrioventricular (AV) block. To avoid right ventricular (RV) pacing, preserving intrinsic AV conduction as much as possible is recommended. However, there is no clear cutoff AV interval to determine whether intrinsic AV conduction should be preserved or RV pacing should be delivered. This study aimed to compare a pacing mode-preserving, intrinsic AV conduction with the DDD mode delivering RV pacing in terms of echocardiographic parameters in patients with first-degree AV block and to investigate whether RV pacing induces heart failure (HF). Stroke volume (SV) was measured to determine the optimal AV delay with the intrinsic AV conduction rhythm and the DDD pacing delivering RV pacing. Echocardiographic evaluation was performed for 6-month follow-up period. Seventeen patients were studied. At baseline, mean intrinsic PQ interval was 250 ± 40 ms. SV was greater with RV pacing with optimal AV delay of 160 ms than with intrinsic AV conduction rhythm in all patients. Therefore, pacemakers were set to the DDD to deliver RV pacing. During follow-up, seven patients developed HF. Mean baseline E/E′ ratio in patients who developed HF (HF group) during RV pacing was higher than in patients without HF (non = HF group; 17.9 ± 8 versus 11.5 ± 2, *P* = 0.018) Even within HF group patients without a high baseline E/E′ ratio, it increased with RV pacing (22.2 ± 6 versus 11.6 ± 2; *P *< 0.001). In patients with pacemaker and first-degree AV block, RV pacing with the optimal AV delay of 160 ms increased SV. However, the risk of HF may be increased with RV pacing if the E/Eʹ ratio is > 15 during intrinsic AV conduction or RV pacing. RV pacing should be avoided in patients with high E/Eʹ ratio under intrinsic AV conduction or RV pacing.

## Introduction

Sick sinus syndrome and advanced atrioventricular (AV) block are often treated with a pacemaker. However, ventricular contraction during right ventricular (RV) pacing might be less efficient than physiological ventricular contraction during intrinsic AV conduction because of dyssynchrony caused by RV pacing [1–4]. Therefore, some studies have suggested that intrinsic AV conduction should be preserved to prioritize physiological ventricular contractions as much as possible [5–7]. Synchronization between the atria and ventricles is also important for cardiac function. In patients with functioning atria (without atrial fibrillation), ventricular contraction with appropriate timing after atrial contraction is an important factor to ensure adequate stroke volume (SV). Although the intrinsic rhythm allows for physiological ventricular contraction, excessive delays in AV conduction time (PR interval) may lead to diastolic mitral regurgitation or adversely affect left ventricular (LV) function. It has been reported that echocardiography is useful for optimizing AV delay settings in patients with pacemakers [8]. AV synchronization is very important for maintaining adequate SV, particularly in patients with heart failure. Therefore, studies have been conducted to explore optimum AV delay for cardiac resynchronization therapy (CRT) [9–11].

Regarding pacemakers for bradycardia, some studies have reported on the optimization of AV delay [8–13]. However, it remains unknown whether intrinsic AV conduction with delays in AV conduction time exceeding physiological levels (corresponding to first-degree AV block) should be preserved or ventricular pacing with AV delay within physiological levels should be delivered to obtain better LV function. RV pacing may cause intraventricular conduction defects or dyssynchrony that leads to impaired LV function. Therefore, this study aimed to compare a pacing mode-preserving, intrinsic AV conduction with the DDD mode delivering RV pacing in terms of echocardiographic parameters in patients with first-degree AV block and to investigate whether RV pacing induces heart failure (HF).

## Materials and methods

### Study design

This prospective observation study was conducted in patients with pacemakers who had an intrinsic rhythm with electrocardiographic findings of first-degree AV block and regularly visited an outpatient clinic for pacemaker checks. The effects of intrinsic AV conduction rhythm with first-degree AV block in the AAI pacing mode and RV paced rhythm with optimal AV delay in the DDD pacing mode on SV were examined. The optimal AV delay to obtain maximum SV was determined under echocardiographic guidance. Patients were followed for 6 months after their pacemaker setting was changed from AAI to DDD pacing mode to determine whether they develop HF, while in the DDD pacing mode with optimal AV delay.

Eighteen patients who had first-degree AV block and without RV pacing (programming either AAI mode or preference intrinsic AV conduction mode) were enrolled in the study between July 2018 and September 2020. One patient was excluded, because atrial fibrillation was detected at screening and a total of 17 patients were studied. Table 1 shows baseline characteristics of enrolled patients. None of study patients had history of HF hospitalization. Two patients had HF symptoms, such as occasional shortness of breath with exercise, and were being treated with medication. The patients underwent blood sampling for N-terminal pro-brain natriuretic peptide (NT-proBNP) testing. They also underwent transthoracic echocardiography. SV was measured (1) under intrinsic AV conduction (PQ interval > 200 ms) and with AV delays of (2) 120 ms, (3) 160 ms, and (4) 200 ms, respectively. The AV delay that provided the maximum SV was considered the optimal AV delay. At the start of the 6-month follow-up period, the pacemakers of all patients were set to DDD pacing mode with an optimal AV delay of 160 ms. The patients were monitored for signs of HF, such as symptom of New York Heart Association class II or more, or pulmonary congestion or increasing cardiothoracic ratio on chest X-ray, and changes in NT-proBNP levels during the follow-up period. This study was approved by the ethics committee of Nihon University Itabashi Hospital. Written informed consent was obtained from all patients (approval number: RK-180313-10).

**Table 1:** Baseline patient characteristics

Age (years)	78 ± 9
Male gender, n (%)	6 (35)
Pacemaker indication	
Sick sinus syndrome, *n* (%)	12 (70)
Transient or advanced atrioventricular block, *n* (%)	5 (30)
AV interval (ms)	250 ± 40
Intrinsic QRS duration (ms)	115 ± 25
Hypertension, *n* (%)	12 (71)
Diabetes mellitus, *n* (%)	1 (6)
Medication	
Beta-blocker, *n* (%)	9 (53)
ACEI/ARB, *n* (%)	11 (65)
Antiarrhythmic agent, *n* (%)	4 (24)
Echocardiographic assessment	
LVDd (mm)	48 ± 4
LVDs (mm)	32 ± 4
Ejection fraction (%)	63 ± 6
Left atrial dimension (mm)	40 ± 8

*ACEI* angiotensin-converting enzyme inhibitor, *ARB*: angiotensin receptor blocker, *AV* atrioventricular, *LVDd* left ventricular diastolic diameter, *LVDs* left ventricular systolic diameter

### Diagnosis of heart failure

The definition of HF was based on the Framingham criteria [14]. The major criteria consisted of paroxysmal nocturnal dyspnea, orthopnea, abnormal jugular venous distention, pulmonary rales, cardiomegaly, pulmonary edema, presence of a third heart sound, central venous pressure > 16 cmH_2_O, and hepatojugular reflex. The minor criteria consisted of edema, night cough, hepatomegaly, pleural effusion, tachycardia > 120 bpm, and weight loss > 4.5 kg in 5 days (considered a major criterion if it occurs during therapeutic interventions for HF). A patient was considered to have HF if two major criteria were present or if one major and two minor criteria were present concurrently. Two cardiologists confirmed the findings in each patient.

### Echocardiographic measurements

Left ventricular ejection fraction (LVEF) and LV mass was calculated with the formula derived from American Society of Echocardiography data [15]. LVEF was generally calculated using the modified Quinones method [16], but the modified Simpson’s method was used in patients with LV dysfunction [17]. LV mass index was calculated as the LV mass-to-body surface area ratio [18]. Transmitral flow velocity curves were recorded to measure peak early diastolic flow velocity (E) and late diastolic flow velocity (A). Tissue Doppler imaging was performed at the level of the septal mitral annulus to measure early diastolic myocardial velocity (E′) and late diastolic myocardial velocity (A′), as previously described. A parasternal long axis view was obtained. The diameter of left ventricular outflow tract (LVOT) was measured during mid-systole. An apical five-chamber view was obtained. Left ventricular outflow tract velocity time integral (LVOT VTI) was measured using pulsed-wave Doppler in the LVOT using a 2 mm sample volume positioned just proximal to the aortic valve. LVOT VTI is considered to be a surrogate of SV according to the following equations:

$$ {\text{Flow rate}} = {\text{cross sectional area}}\left( {{\text{CSA}}} \right) \times {\text{flow velocity and SV}} = {\text{CSA}} \times {\text{VTI}}{.} $$

### Determination of the optimal AV interval in DDD pacing mode

All patients presented with intrinsic AV conduction with a first-degree AV block. At first, echocardiographic assessment including SV was performed with the intrinsic AV conduction rhythm as the baseline. Next, the pacemaker was programmed to the DDD pacing mode while varying the AV delay interval to 120, 160, and 200 ms to determine which setting corresponds to maximum SV. The pacemaker was programmed to the AV delay interval of 160 ms with maximum SV. The setting continued for 6 months, unless cardiac events occurred.

### Statistical analysis

Continuous variables are presented as means ± SD. Categorical variables are expressed as numbers and percentages. Differences between groups were assessed using Student’s *t* test for normally distributed continuous variables and the Mann–Whitney *U* test for non-normally distributed continuous variables. Fisher’s exact test or the chi-squared test was used for categorical variables. *P *< 0.05 was considered to be significant.

## Results

### Study patients

Table 1 shows patient characteristics at baseline. The study included 17 patients with mean age of 78 ± 9 years. There were 6 males (35%) and 11 females (65%). The most common underlying heart disease was sick sinus syndrome (*n* = 12/17; 71%). Mean intrinsic PQ interval was 250 ± 40 ms and mean intrinsic QRS duration was 115 ± 25 ms. Systolic function was preserved in all patients, and mean LVEF was 63 ± 6%. Median NT-proBNP level at screening was 257 pg/dl (interquartile range 105–665 pg/dl).

**Fig. 1:**
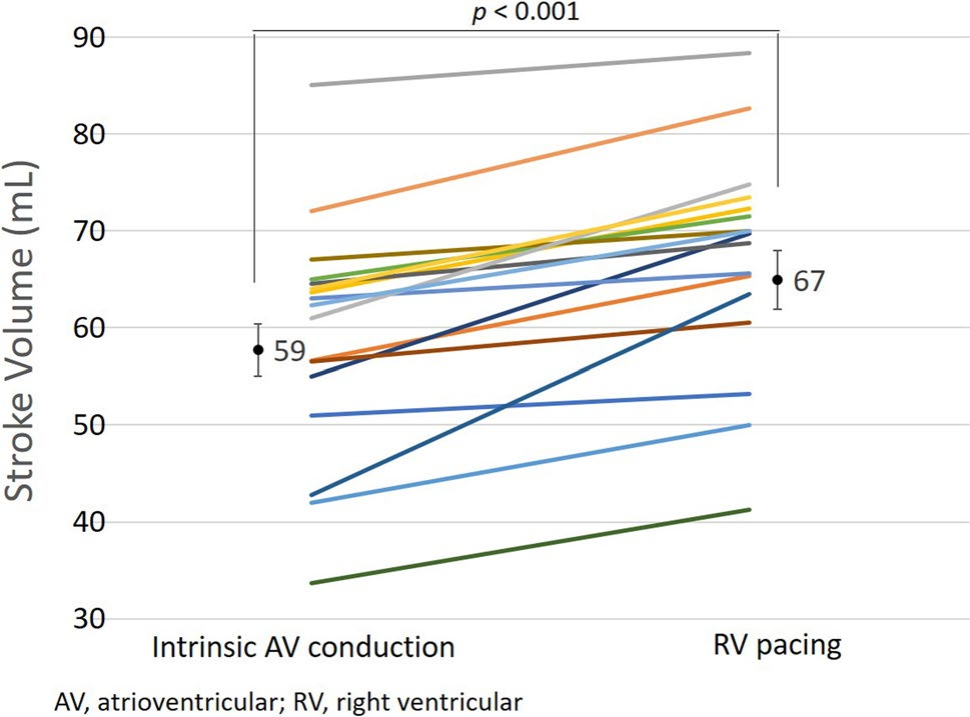
Change in stroke volume with RV pacing. Stroke volume increased with RV pacing at optimal AV delay in all patients. *AV* atrioventricular, *RV* right ventricular

SV during RV pacing with the optimal AV delay of 160 ms was greater than SV during intrinsic rhythm in all patients (59 ± 12 vs. 67 ± 11 ml; *P *< 0.001; Fig. 1). Therefore, at the start of follow-up, pacemakers were set to deliver RV pacing in the DDD pacing mode. For patients who developed HF with RV pacing during the 6-month follow-up period, the pacemaker was re-programmed to the initial setting to preserve intrinsic AV conduction with first-degree AV block whenever possible. We originally planned to enroll many more patients in this study. However, we terminated enrollment when the number of participants reached 17, because the incidence of heart failure was higher than expected.

### Clinical follow-up

**Fig. 2:**
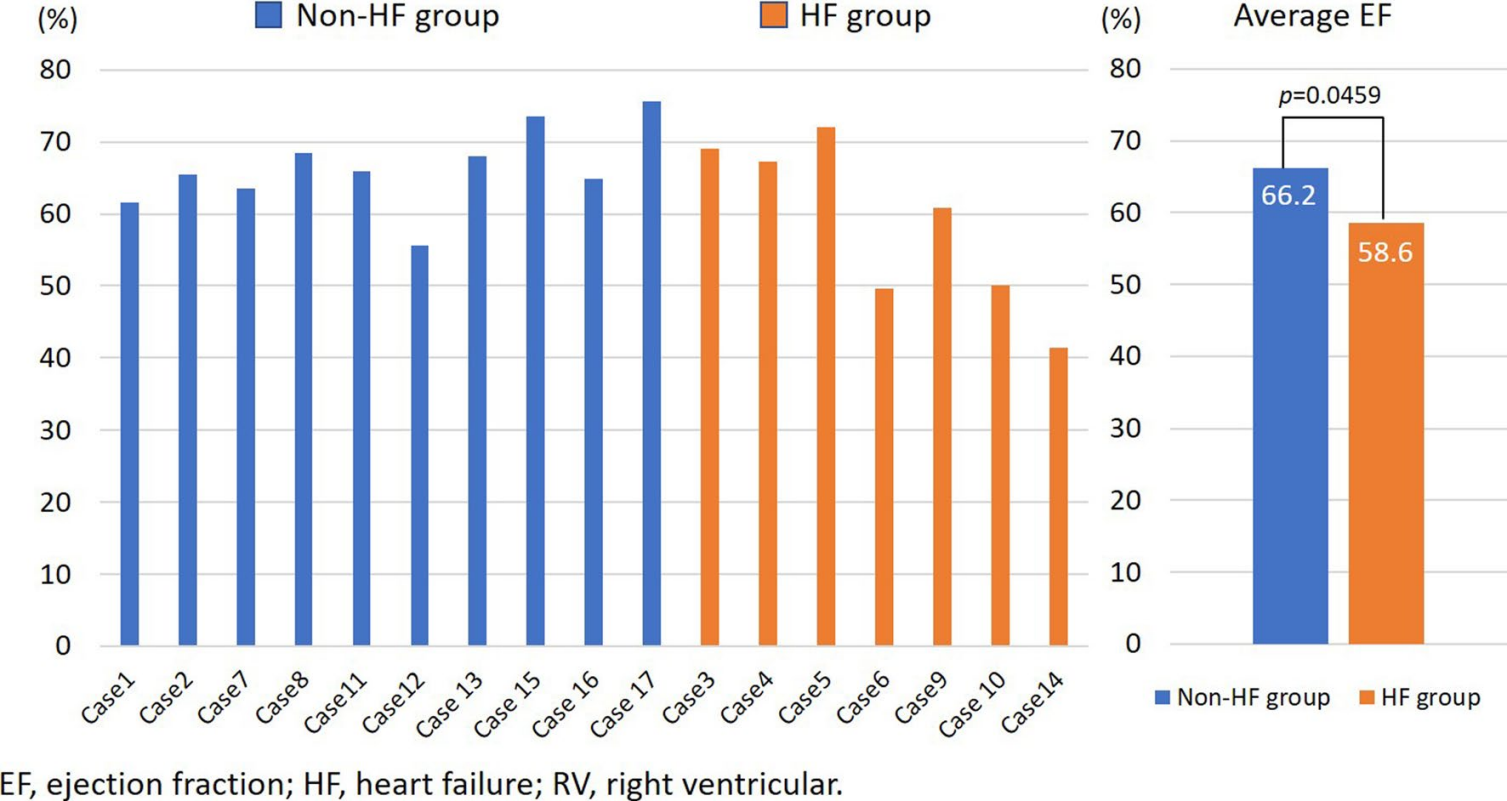
EF with RV pacing at the follow-up echocardiographic assessment by heart failure status. There were no significant differences in EF between the two groups. *EF* ejection fraction, *HF* heart failure, *RV* right ventricular

**Fig. 3:**
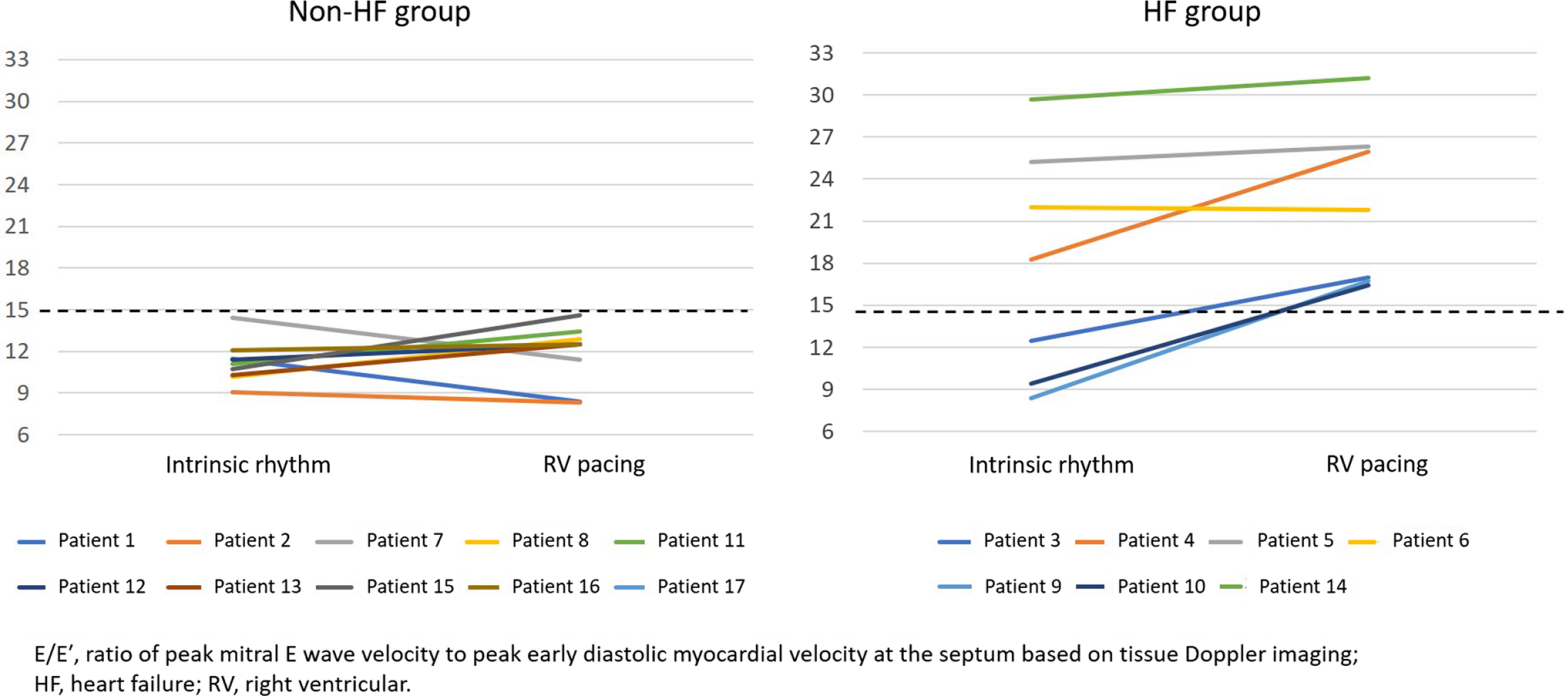
Change in E/Eʹ ratio with RV pacing by heart failure status. Patients in Non-HF group showed low E/E′ ratio both at baseline and after RV pacing. *E/E′* ratio of peak mitral E wave velocity to peak early diastolic myocardial velocity at the septum based on tissue Doppler imaging, *HF* heart failure, *RV* right ventricular

During the 6-month follow-up period with RV pacing, seven patients developed or relapsed into acute decompensated HF. Patients who developed heart failure during the follow-up period were categorized into the heart failure (HF) group. Those who did not develop heart failure were classified into the non-heart failure (non-HF) group. When outcomes were compared, there were no significant differences in baseline electrocardiographic findings and mean QRS duration after the start of RV pacing between the groups (Table 2). Mean NT-proBNP in the HF group was significantly higher than in the non-HF group (166 ± 117 vs. 958 ± 740 pg/dl; *P *= 0.004). Although there was a significant difference in EF at the follow-up assessment between the non-HF and HF groups (66.2% versus 58.6%, *P *= 0.0459), the HF group had relatively preserved EF (Fig. 2). The E/E′ ratio was the only echocardiographic parameter that was significantly higher in the HF group in the AAI pacing mode for preserving intrinsic rhythm (11.0 ± 1.5 vs. 17.9 ± 8.2; *P *= 0.018). Figure 3 shows changes in E/E′ ratio from the intrinsic rhythm phase to the RV pacing phase in both groups. In the non-HF group, no significant changes in E/E′ ratio were observed during intrinsic rhythm or RV pacing. None of the patients had an E/E′ ratio reaching > 15. In the HF group, all patients had E/E′ ratios > 15 during RV pacing.

**Table 2:** Comparisons between the non-heart failure and heart failure groups

	Non-HF group (*n* = 10)	HF group (*n* = 7)	*P* value
Age (years)	77 ± 10	78 ± 8	0.540
Male gender, *n* (%)	4 (40)	2 (28)	0.653
PQ interval (ms)	237 ± 23	267 ± 53	0.126
QRS duration (ms)	116 ± 22	114 ± 32	0.901
Pacing QRS duration (ms)	158 ± 39	141 ± 6	0.364
NT-proBNP (pg/dl)	166 ± 117	957 ± 740	0.004
Stroke volume (ml)	55 ± 10	62 ± 13	0.075
Ejection fraction (%)	64 ± 5	62 ± 7	0.542
E/Eʹ	11.0 ± 1.5	17.9 ± 8.2	0.018
Left atrial dimension (mm)	38 ± 8	44 ± 6	0.072
LV mass index (g/m^2^)	99 ± 16	120 ± 26	0.063
LV relative wall thickness	0.40 ± 0.05	0.40 ± 0.08	0.940

*E/E′* ratio of peak mitral E wave velocity to peak early diastolic myocardial velocity at the septum based on tissue Doppler imaging, *HF* heart failure, *NT-proBNP* N-terminal pro-brain natriuretic peptide, *LV* mass index: left ventricular mass index

Pacemaker settings were re-programmed to prolong AV delay and preserve intrinsic rhythm in all patients in the HF group. However, patients 5, 6, and 10 still required RV pacing, because they did not have intrinsic QRS activity even though the AV delay was prolonged to the maximum possible level. All seven patients in the HF group improved clinically during hospitalization and continued treatment in the outpatient clinic. However, Patients 5 and 6 were re-admitted due to worsening heart failure within the follow-up period. One underwent CRT pacemaker (CRT-P) implantation to enhance treatment. In the non-HF group, no significant changes in E/E′ ratio (11.0 ± 1.5 vs. 11.6 ± 2.1; *P *= 0.44) or NT-proBNP levels (166 ± 117 vs. 153 ± 112 pg/ml; *P *= 0.72) were observed during the follow-up period.

## Discussion

In this study, maximum SV was obtained during RV pacing with optimal AV delay rather than during intrinsic AV conduction in patients with pacemakers and first-degree AV block. However, even though the pacemaker was programmed to the optimal setting based on echocardiographic assessment, seven (41%) patients developed HF, suggesting that some patients may have better hemodynamics in a pacing mode that preserves intrinsic ventricular contraction with very long PR intervals than with RV pacing in the DDD pacing mode with an optimal AV delay, which is believed to be more physiological. This is the first study that compared the incidence of HF between a pacing mode-preserving intrinsic ventricular contraction with prolonged PR intervals and RV pacing with the optimal AV delay in patients with pacemakers.

Sweeney et al. reported that RV pacing induces HF and atrial fibrillation in the Mode Selection Trial (MOST) [9]. The development of HF in seven patients in the present study supports this report. On the other hand, Riahi et al. reported that RV pacing in the DDD pacing mode is not associated with the development of HF [19]. Ten patients in the present study did not develop HF, similar to the results from Riahi et al.

There are many studies indicating that RV pacing is harmful [5–7]. Therefore, pacemaker manufacturers have developed pacemakers with new algorithms that preserve intrinsic conduction. These algorithms include Managed Ventricular Pacing (MVP^™^; Medtronic, Minneapolis, USA) and Ventricular Intrinsic Preference (VIP^™^; Abbott Laboratories, Illinois, USA). In our clinical practice, there have been questions about what is the maximum acceptable prolongation of the PR interval and whether intrinsic AV conduction should be preserved even if the PR interval is ≥ 300 ms when these algorithms are applied. In the present study, RV pacing did not lead to adverse events in approximately 60% of patients. However, RV pacing led to HF in approximately 40% of patients. These findings cannot completely suggest that RV pacing is harmful. In this study, some patients developed HF even though all patients had LVEF > 50% at baseline and their pacemakers were programed with the optimal AV delay to provide the maximum SV, suggesting that the development of HF might not always be associated with impaired systolic function. In contrast, some studies reported that worsening of HF is associated with impaired systolic function [20, 21]. E/E′ ratio has commonly been used as an index of diastolic function in clinical practice and research studies [22, 23]. The present study compared changes in E/E′ ratio before and after RV pacing between the HF and non-HF groups. Compared with the non-HF group, E/E′ ratio worsened to > 15 or increased from baseline in the HF group. Therefore, patients with impaired diastolic function or who develop left heart strain during RV pacing might be at risk of developing HF associated with RV pacing. Although it is difficult to fully explain why the optimal AV delay sometimes leads to HF, we speculate that RV pacing can lead to systolic dyssynchrony despite AV delay optimization, resulting in diastolic dysfunction. In this study, optimal AV delay was determined based on echocardiographic assessment. Echocardiography was performed in the resting state and this programming might not be the best in situations when heart rate increases. Intrinsic AV conduction might differ as heart rate increases. Therefore, it is possible that the optimal AV delay was no longer optimal when the patient is active or in an emotional state. We believe that this is a limitation of AV optimization based on echocardiography, unless the echocardiographic assessment was performed during exercise (e.g., with an ergometer).

### Clinical implications

The results of this study suggested that E/E′ ratio might be useful for predicting an increase in heart strain and the development of heart failure in association with RV pacing. Previous studies have found RV pacing unfavorable and recommended intrinsic conduction [5–7]. However, it is unknown whether a very long AV conduction time is acceptable. In this study, the DDD pacing mode with optimal AV delay resulted in greater SV than very long intrinsic AV conduction based on echocardiographic assessment. Approximately 60% of the study patients did not develop HF during RV pacing, suggesting that the DDD pacing mode with the optimal AV delay, which is believed to be physiological, might be better than intrinsic conduction if the intrinsic AV conduction time is too long. On the other hand, the remaining 40% patients developed HF during RV pacing, indicating that intrinsic conduction should be preferred over RV pacing in such patients even if their intrinsic AV conduction time is substantially longer than the optimal AV delay. These findings might be consistent with findings from the Biventricular versus Right Ventricular Pacing in Heart Failure Patients with Atrioventricular Block (BLOCK HF) trial [24], which showed that CRT should be selected for patients with low cardiac function (LVEF < 50%). This study might help determine whether the current pacemaker should be replaced with CRT-P to enhance treatment. E/E′ ratios before and after RV pacing may be useful information for determining whether intrinsic AV conduction should be preferred or the current pacemaker should be replaced with CRT-P early in patients with first-degree AV block who will inevitably require pacing.

### Limitations

This study has some limitations. First, the sample size was small, with only 17 patients. We originally planned to include more patients, but had to discontinue patient enrollment, because some patients developed HF associated with RV pacing and there was a possible risk of causing a disadvantage to patients. Second, follow-up duration was too short to determine the effect of RV pacing on HF, due to the same reason as for the first limitation. However, despite this short follow-up duration, a relatively high number of patients developed HF unexpectedly. This result demonstrated that undesirable effects are possible with RV pacing in certain patients. Third, the AV delay that provided the maximum SV was selected as the optimal AV delay based only on echocardiography. We are not sure whether relying only on echocardiographic assessment is the gold standard. However, we selected this strategy because previous studies also set AV delay based on echocardiographic findings [8–13].

## Conclusions

In patients with pacemakers and first-degree AV block, RV pacing with the optimal AV delay generally increased SV, but it should be used carefully because RV pacing may cause HF in some patients. E/E′ ratio may be useful for predicting the development of HF associated with RV pacing.

## Data Availability

The data in this study are available on request from the corresponding author.

## References

[1] Lee MA, Dae MW, Langberg JJ, Griffin JC, Chin MC, Finkbeiner WE, O’Connell JW, Botvinick E, Scheinman MM, Rosenqvist M (1994). Effects of long-term right ventricular apical pacing on left ventricular perfusion, innervation, function and histology. J Am Coll Cardiol.

[2] Zhang XH, Chen H, Siu CW, Yiu KH, Chan WS, Lee KL, Chan HW, Lee SW, Fu GS, Lau CP, Tse HF (2008). New-onset heart failure after permanent right ventricular apical pacing in patients with acquired high-grade atrioventricular block and normal left ventricular function. J Caldiovasc Electrophysiol.

[3] Thackray SDR, Witte KKA, Nikitin NP, Clark AL, Kaye GC, Cleland JGF (2003). The prevalence of heart failure and asymptomatic left ventricular systolic dysfunction in a typical regional pacemaker population. Eur Heart J.

[4] Ogano M, Tsuboi I, Iwasaki Y, Tanabe J, Shimizu W (2021). Structural heart disease, not the right ventricular pacing site, determines the QRS duration during right ventricular pacing. Heart Vessels.

[5] Chan WY, Blomqvist A, Melton IC, Norén K, Crozier IG, Benser ME, Eigler NL, Gutfinger D, Troughton RW (2014). Effects of AV delay and VV delay on left atrial pressure and waveform in ambulant heart failure patients: insights into crt optimization. Pacing Clin Electrophysiol.

[6] Fukuhara K, Okura H, Koyama T, Kume T, Neishi Y, Hayashida A, Yoshida K (2015). Feasibility of a novel atrioventricular delay optimization method using transmitral and pulmonary venous flow in patients with sequential ventricular pacing or cardiac resynchronization therapy. J Echocardiogr.

[7] Chatterjee NA, Gold MR, Waggoner AD, Picard MH, Stein KM, Yu Y, Meyer TE, Wold N, Ellenbogen KA, Singh JP (2016). Longer left ventricular electric delay reduces mitral regurgitation after cardiac resynchronization therapy: Mechanistic insights from the smart-av study (smartdelay determined av optimization: a comparison to other av delay methods used in cardiac resynchronization therapy). Circ Arrhythm Electrophysiol.

[8] Ishikawa T, Sumita S, Kimura K, Kikuchi M, Kosuge M, Kuji N, Endo T, Sugano T, Sigemasa T, Kobayashi I, Tochikubo O, Usui T (1999). Prediction of optimal atrioventricular delay in patients with implanted DDD pacemakers. Pacing Clin Electrophysiol.

[9] Sweeney MO, Hellkamp AS, Ellenbogen KA, Greenspon AJ, Freedman RA, Lee KL, Lamas GA (2003). Adverse effect of ventricular pacing on heart failure and atrial fibrillation among patients with normal baseline qrs duration in a clinical trial of pacemaker therapy for sinus node dysfunction. Circulation.

[10] Sharma AD, Rizo-Patron C, Hallstrom AP, O'Neill GP, Rothbart S, Martins JB, Roelke M,Steinberg JS, Greene HL, DAVID Investigators (2005). Percent right ventricular pacing predicts outcomes in the DAVID trial. Heart Rhythm.

[11] Sweeney MO, Bank AJ, Nsah E, Koullick M, Zeng QC, Hettrick D, Sheldon T, Lamas GA, Search AV Extension and Managed Ventricular Pacing for Promoting Atrioventricular Conduction (SAVE PACe) Trial (2007). Minimizing ventricular pacing to reduce atrial fibrillation in sinus-node disease. N Engl J Med.

[12] Ishikawa T, Sugano T, Sumita S, Nakagawa T, Hanada K, Kosuge M, Kobayashi I, Kimura K, Tochikubo O, Usui T, Umemura S (2001). Optimal atrioventricular delay setting determined by evoked QT interval in patients with implanted stimulus-T-driven DDDR pacemakers. Europace.

[13] Kato M, Dote K, Sasaki S, Goto K, Takemoto H, Habara S, Hasegawa D, Matsuda O (2005). Determination of the optimal atrioventricular interval in sick sinus syndrome during DDD pacing. Pacing Clin Electrophysiol.

[14] McKee PA, Castelli WP, McNamara PM, Kannel WB (1971). The natural history of congestive heart failure: the Framingham study. N Engl J Med.

[15] Takeda Y, Sakata Y, Higashimori M, Mano T, Nishio M, Ohtani T, Hori M, Masuyama T, Kaneko M, Yamamoto K (2009). Noninvasive assessment of wall distensibility with the evaluation of diastolic epicardial movement. J Card Fail.

[16] Quinones MA, Waggoner AD, Reduto LA, Nelson JG, Young JB, Winters WL, Ribeiro LG, Miller RR (1981). A new, simplified and accurate method for determining ejection fraction with two-dimensional echocardiography. Circulation.

[17] Vuille C, Wayman AE, Weyman AE (1994). Left ventricle I: general considerations, assessment of chamber size and function. Principles and practice of echocardiograpy.

[18] Aizawa Y, Sakata Y, Mano T, Takeda Y, Ohtani T, Tamaki S, Omori Y, Tsukamoto Y, Hirayama A, Komuro I, Yamamoto K (2011). Transition from asymptomatic diastolic dysfunction to heart failure with preserved ejection fraction: roles of systolic function and ventricular distensibility. Circ J.

[19] RiahiS, Nielsen JC, Hjortshøj S, Thomsen PEB, Højberg S, Møller M, Dalsgaard D, Nielsen T, Asklund M, Friis EV, Christensen PerD, Simonsen EH, Eriksen UH, Jensen GVH, Svendsen JH, Toff WD, Healey JS, Andersen HR, DANPACE Investigators (2012). Heart failure in patients with sick sinus syndrome treated with single lead atrial or dual-chamber pacing: no association with pacing mode or right ventricular pacing site. Europace.

[20] Redfield MM, Jacobsen SJ, Burnett JC, Mahoney DW, Bailey KR, Rodeheffer RJ (2003). Burden of systolic and diastolic ventricular dysfunction in the community: appreciating the scope of the heart failure epidemic. JAMA.

[21] Kane GC, Karon BL, Mahoney DW, Redfield MM, Roger VL, Burnett JC, Jacobsen SJ, Rodeheffer RJ (2011). Progression of left ventricular diastolic dysfunction and risk of heart failure. JAMA.

[22] Nagueh SF, Appleton CP, Gillebert TC, Marino PN, Oh JK, Smiseth OA, Waggoner AD, Flachskampf FA, Pellikka PA, Evangelista A (2009). Recommendations for the evaluation of left ventricular diastolic function by echocardiography. J Am Soc Echocardiogr.

[23] McMurray JJV, Adamopoulos S, Anker SD, Auricchio A, Böhm M, Dickstein K, Falk V,Filippatos G, Fonseca C, Gomez-Sanchez MA, Jaarsma T, Køber L, Lip GYH, Maggioni AP, Parkhomenko A, Pieske BM, Popescu BA, Rønnevik PK, Rutten FH, Schwitter J, Seferovic P, Stepinska J, Trindade PT, Voors AA, Zannad F, Zeiher A, ESC Committee for Practice Guidelines (2012). ESC guidelines for the diagnosis and treatment of acute and chronic heart failure 2012: the task force for the diagnosis and treatment of acute and chronic heart failure 2012 of the European Society of Cardiology. Developed in collaboration with the Heart. Eur Heart J.

[24] Curtis AB, Worley SJ, Adamson PB, Chung ES, Niazi I, Sherfesee L, Shinn T, Sutton MS, Biventricular versus Right Ventricular Pacing in Heart Failure Patients with Atrioventricular Block (BLOCK HF) Trial Investigators (2013). Biventricular pacing for atrioventricular block and systolic dysfunction. N Engl J Med.

